# A comparison of nucleosome organization in *Drosophila* cell lines

**DOI:** 10.1371/journal.pone.0178590

**Published:** 2017-06-01

**Authors:** Rebecca L. Martin, John Maiorano, Greg J. Beitel, John F. Marko, Graham McVicker, Yvonne N. Fondufe-Mittendorf

**Affiliations:** 1 Department of Molecular Biosciences, Northwestern University, Evanston, Illinois, United States of America; 2 Department of Molecular and Cellular Biochemistry, University of Kentucky, Lexington, Kentucky, United States of America; 3 Department of Physics and Astronomy, Northwestern University, Evanston, Illinois, United States of America; 4 Salk Institute for Biological Studies, La Jolla, California, United States of America; Saint George's University, UNITED KINGDOM

## Abstract

Changes in the distribution of nucleosomes along the genome influence chromatin structure and impact gene expression by modulating the accessibility of DNA to transcriptional machinery. However, the role of genome-wide nucleosome positioning in gene expression and in maintaining differentiated cell states remains poorly understood. *Drosophila melanogaster* cell lines represent distinct tissue types and exhibit cell-type specific gene expression profiles. They thus could provide a useful tool for investigating cell-type specific nucleosome organization of an organism’s genome. To evaluate this possibility, we compared genome-wide nucleosome positioning and occupancy in five different Drosophila tissue-specific cell lines, and in reconstituted chromatin, and then tested for correlations between nucleosome positioning, transcription factor binding motifs, and gene expression. Nucleosomes in all cell lines were positioned in accordance with previously known DNA-nucleosome interactions, with helically repeating A/T di-nucleotide pairs arranged within nucleosomal DNAs and AT-rich pentamers generally excluded from nucleosomal DNA. Nucleosome organization in all cell lines differed markedly from *in vitro* reconstituted chromatin, with highly expressed genes showing strong nucleosome organization around transcriptional start sites. Importantly, comparative analysis identified genomic regions that exhibited cell line-specific nucleosome enrichment or depletion. Further analysis of these regions identified 91 out of 16,384 possible heptamer sequences that showed differential nucleosomal occupation between cell lines, and 49 of the heptamers matched one or more known transcription factor binding sites. These results demonstrate that there is differential nucleosome positioning between these *Drosophila* cell lines and therefore identify a system that could be used to investigate the functional significance of differential nucleosomal positioning in cell type specification.

## Introduction

Over 75% of eukaryotic DNA within a nucleus is compacted into chromatin fibers that contain long repeating arrays of nucleosomes. In each nucleosome unit, a segment of DNA is wrapped around a histone protein core [1]. An essential role of chromatin is to compact the large amount of genomic DNA into the confines of the eukaryotic nucleus, but nucleosomes also physically occlude DNA from interactions with other DNA binding proteins [2–4]. Thus, the nucleosome structure is considered to be repressive to gene expression [5, 6]. Indeed, depleting nucleosomes in yeast activates previously repressed genes even in the absence of activating transcription factors [7]. Controlled changes in nucleosome placement along the DNA are predicted to have regulatory roles in gene transcription [8–10]. Furthermore, the competition between nucleosomes and transcription factors for binding to the DNA strand can be considered an additional layer of epigenetic regulation of gene expression [11–14]. Because transcription factor concentration and access to genetic information changes with growth, cell differentiation and in response to environmental stimuli, the chromatin organization and nucleosome positioning must also change rapidly and precisely.

Positioning of nucleosomes is directed by two major factors: intrinsic DNA-histone interactions, and positioning of nucleosomes by remodeling complexes [15–22]. For most nucleosomes, each nucleosome is a discrete unit consisting of 147 base pairs (bp) of DNA wrapped around a histone octamer; 2 pairs of histones H2A H2B, and 2 pairs of H3 and H4 [23]. Previous work demonstrated that DNA sequences wrapped around a nucleosome exhibit predictable patterns that influence nucleosome occupancy [24–27]. In particular, the histone octamer prefers placement along DNAs containing 10 base pair repeats of AA/AT/TT dinucleotides out of phase with CG dinucleotide repeats [28–30]. The phased helical repeats of A/T dinucleotides every 10 base pairs allow for flexion of nucleosomal DNA around the histone octamer. Furthermore, poly-A kmers are generally excluded from nucleosomal DNA. Acting on top of the biochemical interactions that drive nucleosome positioning, the positions of nucleosomes can be altered by chromatin remodeling complexes [31, 32]. These factors should therefore direct the landscape of nucleosome occupancy that characterizes a specific cell state following differentiation.

Previously, cell differentiation was considered to be driven solely by controlled expression of transcription factors (TFs) [33–38]. However, it is now recognized that cell fate depends not only on the expression of TFs, but also on the accessibility of target sites within the genome [4, 11, 39, 40]. During differentiation, access to promoters of genes involved in cell-type specific transcription requires rearrangement of nucleosomes over and around particular transcription factor binding sites (TFBS) [13]. Recent studies have described physical changes to chromatin, including epigenetic changes, in specific loci that mark cell fate [11, 41, 42]. However, to fully understand the role of nucleosome positioning in cell-type determination, it is essential to conduct genome-wide analyses of nucleosome occupancy in different cell types. Genome-wide studies have been performed, but predominately in whole multicellular, multistage organisms [43]. Because the concentration of histones, and therefore the number, positioning and occupancy of nucleosomes, differs between different cell types, and during developmental stages, use of whole organisms may obscure underlying patterns of organization. We therefore, decided to examine nucleosome positioning and occupancy in different tissue lineages represented by the standard Drosophila S2 cell line and four distinct *Drosophila* L3 imaginal disc cell lines: leg, eye, antennal and haltere [44].

*Drosophila melanogaster* is an attractive model to use because the relatively small genome of the organism allows for reasonable coverage of mapped reads during parallel sequencing. The various cultured *Drosophila* cell lines are extensively characterized and therefore provide a powerful model for understanding cell-type specification. Several studies have characterized the unique differential expression profiles for many of the available *Drosophila* cell lines [33, 45]. While each cell line necessarily possesses the same genome, each line maintains a distinct transcriptional profile that represents its tissue source and the concentration of factors driving expression [33, 34].

In this work, we compare nucleosome positioning over key genomic regions and DNA sequences in distinct *Drosophila* cell lines. We report *in vivo* nucleosome positioning maps for the standard S2 cell line that is of embryonic hemocyte origin, and for the antennal, eye, haltere and leg cell lines that are derived from imaginal discs. We characterized patterns of differential coverage by examining nucleosome occupancy and positioning throughout the genome. By comparing nucleosome maps to each other and to the map from *in vitro* reconstituted *Drosophila* chromatin, we uncovered differences from intrinsic nucleosome organization that correlate with possible binding sites for *in vivo* factors that may direct cell type specification.

## Materials and methods

### *Drosophila* cell culture

The following *D*. *melanogaster* cell lines were obtained from the *Drosophila* Genomics Resource Center—DGRC (https://dgrc.cgb.indiana.edu/). The five cell lines used in this study were: S2 (late embryonic cell line); Cme-L1 (leg disc imaginal cell line); ML-DmD11 (eye-antennal disc cell line); ML-DmD20 (antennal disc cell line); and the ML-DmD17 (haltere disc cell line). Cells were cultured in the DGRC recommended culture media at 24°C. S2 cells were cultured in Schneider's *Drosophila* medium (Invitrogen) supplemented with 10% FCS (Hyclone); Cme-L1 cells were maintained in M3 (Sigma-Aldrich), supplemented with 2% FCS, 5 μg/ml insulin (Sigma), and 2.5% fly extract, while ML-DmD11, ML-DmD20 and ML-DM17 cells were maintained in M3+BPYE supplemented with 10% FCS and 10 μg/ml insulin. For the experiments, cells were replated on 60 mm plastic dishes at a density of 0.5–1×10^6^ cells/ml, and allowed to proliferate for 3–4 days until they became they reached ~85% confluency. Cell harvest required only gentle agitation to dislodge the semi-adherent cells, which were pelleted by centrifugation at 1200 x g then washed three times with PBS.

### *In vivo* mononucleosome purification

~100 million cells were collected from healthy cell cultures, pelleted and washed with ice-cold PBS. The cell lines were cultured in parallel in identical conditions and digested samples were combined after bar coding, but before sequencing. Cells were resuspended in NP-40 lysis buffer (10 mM Tris-Cl, pH 7.4; 10 mM NaCl; 3 mM MgCl_2_; 0.5% NP-40; 0.15 mM spermine; 0.5 mM spermidine). PMSF and BZA (Sigma) were added to final concentrations of 1 mM and 0.4 mM respectively. Cells were lysed by a 5-minute incubation on ice, the nuclei were pelleted and then washed once with PBS. After gentle resuspension in MNase digestion buffer (10 mM Tris-Cl, pH 7.4; 15 mM NaCl; 60 mM KCl; 0.15 mM spermine; 0.5 mM spermidine; 1 mM CaCl_2_), chromatin was digested with Micrococcal nuclease (Sigma N3755) for 10 minutes at room temperature. Digestion was stopped with MNase stop solution (0.25 M EDTA, 5% SDS added to a final ratio of 1:10 buffer volume) and 5 M NaCl (added to a ratio of 1:5 buffer volume). MNase-digested DNA was isolated from histones and other DNA binding proteins by phenol/chloroform extraction and ethanol precipitation. 10 mg/mL RNAse was added and the purified DNA was incubated for 30 minutes at 37°C to remove any residual RNA.

Digested DNA was sized by running on a 3% agarose gel (NuSieve Lonza). Nucleosomal DNA bands were visualized by UV illumination and mononucleosomal DNA (mnDNA) corresponding in size to 150 bp was excised from the gel. mnDNA was recovered by a mild “crush and soak” protocol [17]. Briefly, excised gel slices were covered in crush and soak buffer (300 mM NaOAc and 1mM EDTA, pH 8.0), and crushed with a microtube pestle inside the centrifuge tube. The gel and buffer slurry was then incubated at room temperature for 48 hours on a bench rocker to allow DNA to passively diffuse into the buffer. Solubilized DNA was separated from the agarose using spin-filters (Amicon Ultrafree-CL filter), centrifuged at 5000 g for 3 minutes and purified (QIAquick PCR purification kit, Qiagen 28104). The DNA was then prepared for ABI SOLiD sequencing following the standard ABI protocols [43].

### Genomic DNA purification and *in vitro* reconstitution of chromatin

To obtain histone octamers for *in vitro* reconstitutions, chicken erythrocytes were prepared as described previously [25]. Briefly, histone octamer and purified genomic DNA from S2 cells were mixed at a 0.8:1 molar ratio in reconstitution buffer (2 M NaCl; 5mM Tris; 1mM benzolamide; 0.5 mM PMSF; 0.5 mM EDTA) and loaded into a 12–14 kDa, 10 mm diameter dialysis tubing, which was then placed into a larger 6–8 kDA 100 mm dialysis bag filled with 100mL of reconstitution buffer. This assembly was then dialyzed against 4 liters of low salt dialysis buffer (5mM Tris; 1mM benzolamide; 0.5mM PMSF; 0.5mM EDTA) at 4°C for a minimum of 24 hours. After 24 hours the 4 liters of cold dialysis buffer were replaced and dialyzed for an additional 24 hours, and the process repeated for a total of 5 dialysis incubations. Reconstituted chromatin was then digested with MNase as described and prepared for ABI SOLiD sequencing generating 27,542,643 unique read pairs.

### SOLiD sequencing, read mapping and analysis

For sequencing, nucleosomal DNA fragments were gel-extracted, end-repaired (End-it-DNA End-Repair kit; Epicentre) and ligated to adaptors using the recommended ABI SOLiD Fragment Library reagents and protocol (Applied Biosystems PN 4464412). The DNA fragments were amplified by PCR for 10 cycles or less prior to ABI SOLiD sequencing. PCR fragments were purified and loaded on a SoLiD flow cell for cluster generation. Nucleosomal reads were separated into separate library files based on their barcodes, and mapped to the *Drosophila* dm3 reference genome using the ABI BioScope^™^ software (Applied Biosystems). SOLiD sequencing generated 4 million to 12 million uniquely mapped reads for each sample. From the aligned reads, only unique, paired DNA fragments sized between 101 and 191 bp were retained for use in the analysis dataset. Nucleosome fragment length was estimated as the distance between paired reads and the midpoint of each mapped fragment was considered the nucleosome midpoint. To generate AA/AT/TA/TT and CC/CG/GC/GG frequency plots, we extracted dinucleotide counts surrounding every nucleosome midpoint. We then computed the frequency of d:A/d:T and d:C/d:G dinucleotides at each distance from the nucleosome midpoint. One sample from each cell line or in vitro chromatin reconstitution was prepared and sequenced. The samples were processed in parallel, and a high degree of similarity in nucleosome occupancy was observed between cell lines (R values > 0.99 for heptamer coverage in each cell line compared to mean combined rate and R value = 0.91 for *in vitro* compared to mean combined rate as described in results) and observed in nucleosome profiles shown in S2 Fig.

The gene sets and annotations used in these analyses were from FlyBase BDGP Release 5. RNA-seq reads from S2 cells were obtained from modENCODE [33]. The number of RNA-seq reads that overlapped with annotated exons in each transcript were counted and normalized by transcript length to obtain fragments per kilobase per million mapped reads (FPKM). Analyses used the log_10_ FPKM value as the expression measurement.

## Results

### Canonical nucleosome positioning sequence features are maintained in all cell lines

Since each *Drosophila* cell line in our study (Table 1) contains the same genomic DNA, we first determined the extent to which the positions of nucleosomes in each cell line are defined by expected nucleosome positioning signals. Previous studies have demonstrated that the positioning of nucleosomes is influenced by the genome sequence [2]. The underlying DNA can influence both the translational position, where the nucleosome ‘sits’ along a stretch of DNA sequence, as well as the rotational position of the DNA around the histone octamer. In the latter case, repeating AA/TA/TT dinucleotide pairs, positioned every 10 bp, or one helical turn, coupled with an out-of-phase 5 bp GG/GC/CC/CG pattern, present highly favorable locations for nucleosome occupancy [3, 29, 30, 46, 47]. It is thought that these DNA sequences have an increased flexibility that allows wrapping around the histone octamer. In contrast, long stretches of adenosine nucleotides, poly-A kmers, resist DNA bending and create unfavorable landscapes for nucleosome positioning, thus influencing the nucleosome translational position [48, 49].

**Table 1:** *Drosophila* cell lines used in this study and number of sequenced paired-end reads mapped to *Drosophila* genome for each cell line.

Cell line (short name)	tissue source	Unique mapped paired reads
ML-DmD20-c2 (D20-c2)	Antennal, L3 disc	8,407,938
ML-DmD11 (D11)	Eye-antennal, L3 disc	4,124,257
ML-DmD17-c3 (D17-c3)	Haltere, L3 disc	9,621,317
CME-L1 (L1)	Leg, L3 disc	11,888,602
S2	Hematocyte, embryo	25,092,601

To determine if the C/G and A/T nucleosome-positioning signals are present in the cell lines used in this study, we collected nucleosomal fragments from them and sequenced them using ABI’s SOLiD paired-end sequencing technique. Deep sequencing produced 4–12 million reads for each cell line (Table 1). We retained only read pairs that mapped uniquely to the *Drosophila* reference genome, with a separation of between 101 bp and 191 bp. The fragments retained and used for analysis are correspond well to the expected lengths for mononucleosomes with mean and median values close to 147bp (S1 Fig). We used the midpoint between the mapped reads as an estimate of the nucleosome midpoint (i.e. dyad) position. As detailed below, the nucleosome profiles for each cell line correlate well with one another, and with previously published data. In addition, nucleosome plots of arbitrary genomic regions show typical occupancy profiles (S2 Fig). Importantly, while some occupancy peaks are shared between all the cell lines and the *in vitro* chromatin (black boxes, S2 Fig, see below for description of in vitro chromatin preparation), other peaks are only shared between cell lines but are greatly reduced or much more substantial in the *in vitro* chromatin (red boxes, S2 Fig).

**Fig 1:**
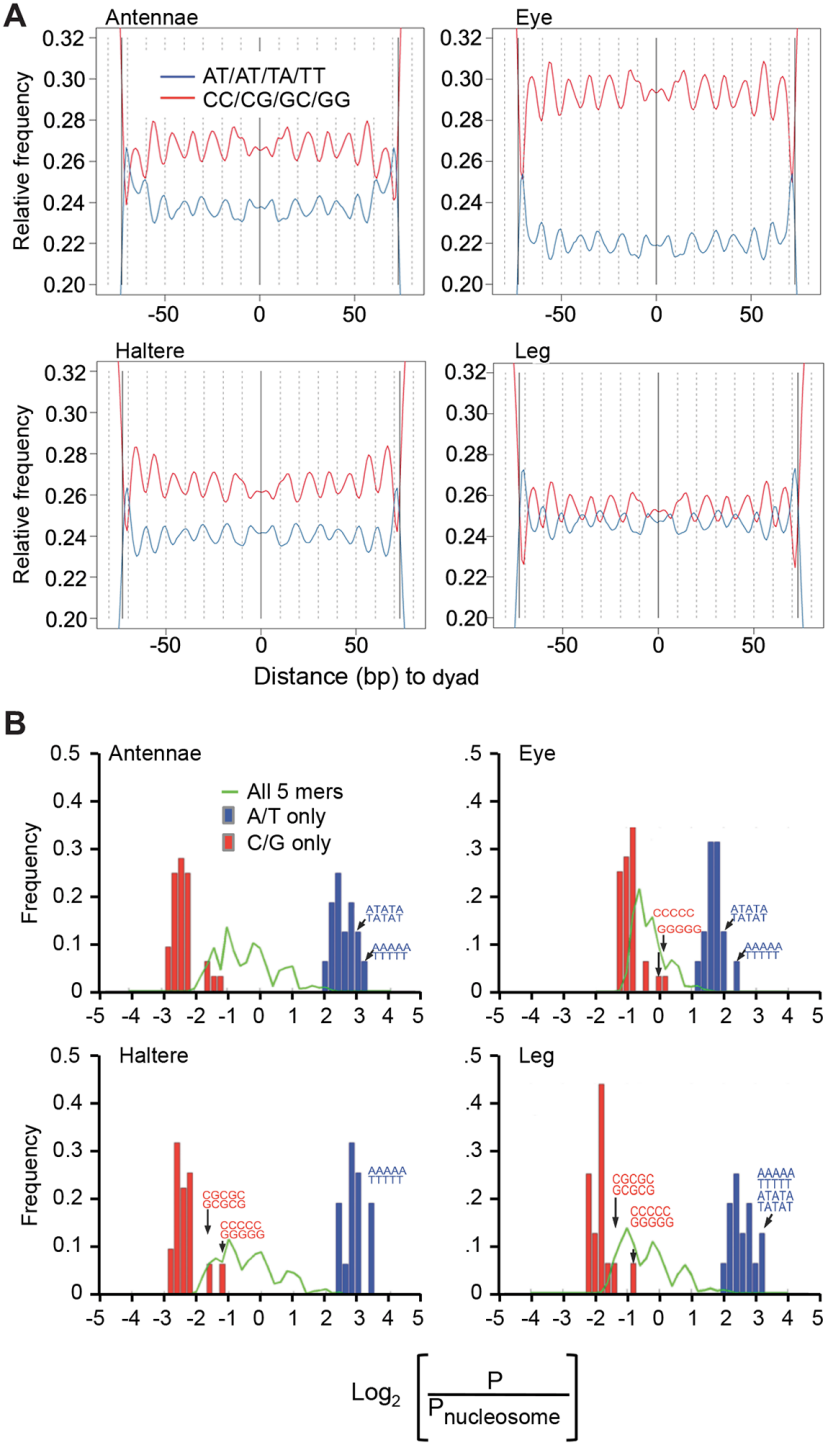
Nucleosomal DNA from *Drosophila* cell lines contains helically repeating dinucleotide patterns and excludes A/T rich pentamers. (A) The relative frequencies of occurrences of AA/AT/TA/TT dinucleotides (blue line) and CC/CG/GC/GG (red line) dinucleotides found at each location from the nucleosome dyad outward are shown for antennae, eye, haltere and leg cell lines. The expected repeating pattern of 10 bp offset dinucleotides, seen in nucleosome studies of *Drosophila* and other organisms, is observed repeating from the center of the nucleosome dyad outward. (B) Distributions of log_2_ frequency ratios for different sets of pentamers. For each pentamer the log_2_(P/P_nucleosome_) was computed, where P is the frequency of the pentamer in the genome, and P_nucleosome_ is the frequency of the pentamer in nucleosomal DNA. Negative values indicate that a pentamer is more frequent within nucleosomal DNA than expected given the frequency of the pentamer in the genome. Separate distributions of log_2_(P/P_nucleosome_) are plotted for the 32 pentamers that contain only A and T (blue); the 32 pentamers that contain only G and C (red); and the complete set of all 1024 pentamers (green). Example pentamer sequences are noted in each plot. In all cell lines, A- and/or T-only pentamers (blue) are excluded from nucleosomal DNA whereas C- and/or G-only pentamers (red) are found preferentially within nucleosomal DNA.

We examined the frequency of dinucleotides along the 147 bp surrounding nucleosome midpoints in aggregate, and found that sequenced reads from each cell line exhibit the helically repeating AA/TA/TT pattern (Fig 1A), as has been observed in *Drosophila* [23, 34]. Further, nucleosome disfavoring poly(dA:dT) tracts tend to be excluded from nucleosomal DNA (Fig 1B). Our data demonstrate that each cell type retains the expected larger organizational nucleosome-positioning signals that influence rotational and translational placement.

### Nucleosome organization surrounding transcription start sites is correlated with levels of gene expression

Nucleosomes have a well-defined configuration in promoter regions, which has been observed in many organisms [18, 19, 27, 46, 50, 51]. This configuration consists of a nucleosome-depleted region (NDR) upstream of a strongly positioned +1 nucleosome. Establishment of the NDR at the TSS is important for regulation of gene expression [32, 51–53]. The +1 nucleosome is followed by an array of nucleosomes downstream, that become less well positioned as distance from the transcription start sites (TSS) increases. Furthermore, analysis of chromatin from several organisms, including *Drosophila*, reveal that phasing of the nucleosome array downstream of the TSS corresponds with gene expression and that genes with high expression have more regularly spaced nucleosome arrays than low expression genes [19, 22, 54–56]. During increased transcriptional activity rapid dynamic rearrangement of this pattern occurs [13, 57].

**Fig 2:**
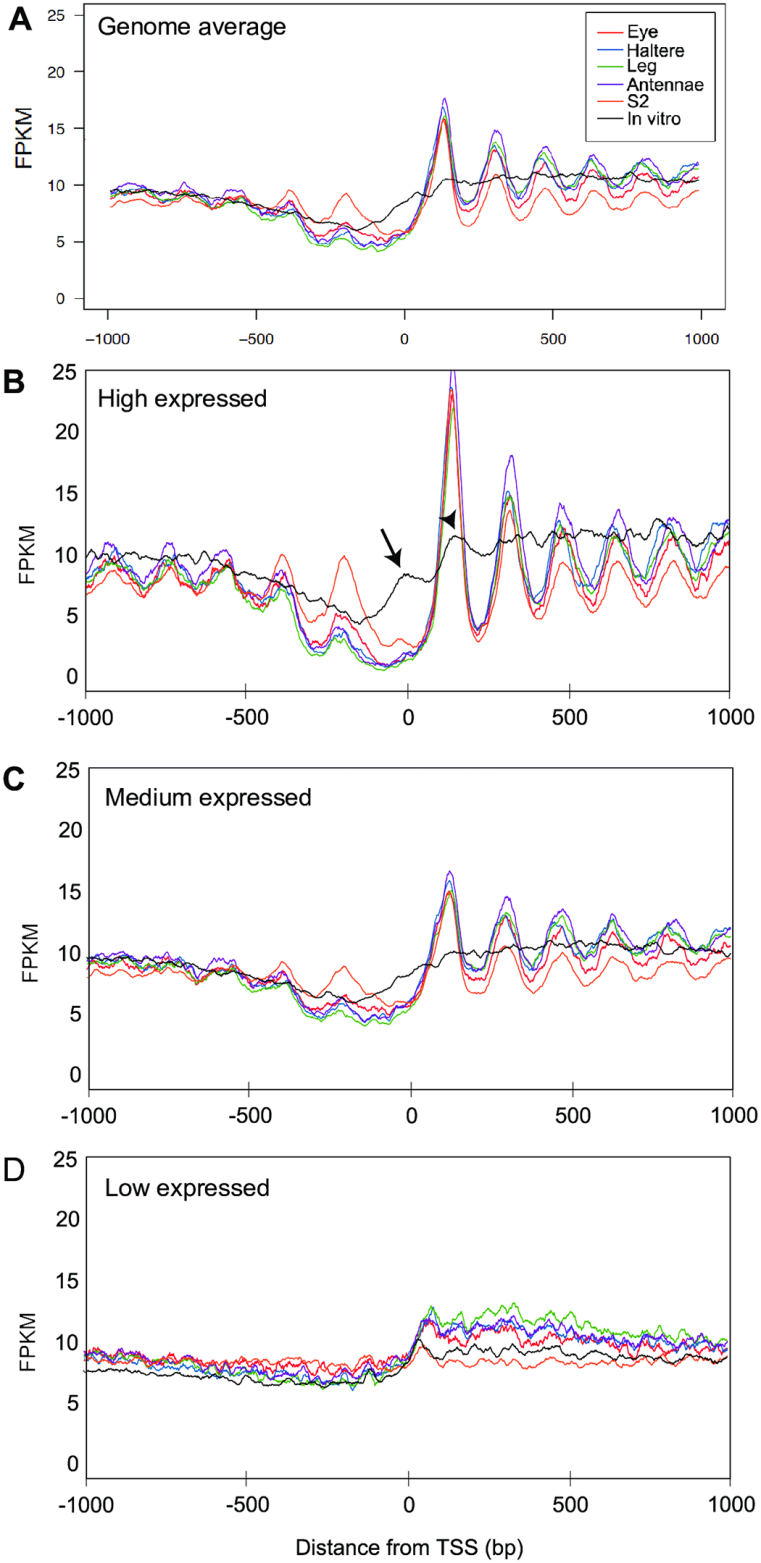
The strength of nucleosome positioning surrounding the TSS in *Drosophila* cell lines is correlated with gene expression level. (A) The rate of nucleosomal midpoints in each cell line was calculated for 1kb upstream and 1kb downstream of known unique transcription start sites (TSSs) of genes in the *Drosophila* genome. The expected pattern is observed where there is a strong +1 nucleosome upstream of the TSS followed by nucleosomes positioned with decreasing strength. (B-D). The fragments per kilobase per million mapped reads (FPKM) of each nucleosome was plotted relative to the TSS in high-expression genes (B, highest 25% of genes), in medium-expression genes (C, central 50%), and low-expression genes (D, lowest 25%). RNA-seq data was obtained from modENCODE [33]. Each plot shows MNase midpoints from fragments in the range of 101–191 bp, smoothed with a 20 bp sliding window. In addition, data for *in vitro* reconstitution of *Drosophila* chromatin are shown, which to some extent mimic some of the features of the cell-line nucleosome positioning data.

We asked to what extent this promoter organization is maintained and reproducible between the cell lines used in this study. We aggregated nucleosome midpoints across all annotated TSSs and found that all cell lines exhibit the expected nucleosome configuration around TSSs (Fig 2A).

We next asked how nucleosome organization correlates with gene expression in these cell lines by partitioning genes into low, medium and high expression groups (bottom 25%, middle 50% and top 25%, respectively). Genes with medium and high expression show a well-positioned nucleosome configuration around the TSS (Fig 2B and 2C). In contrast, genes with low expression do not show a pattern of well-positioned nucleosomes (Fig 2D). These results are consistent with the nucleosome maps previously observed in whole embryos [56] but our results extend these observations to differentiated homogenous cell lines. These results are also consistent with a lack of consistent nucleosome organization in low expression genes in both lower and higher eukaryotes [27, 56, 58, 59].

In yeast, worms, flies and humans the NDR has been observed even in the absence of DNA binding proteins, and therefore could be attributed to the underlying DNA sequence [2, 8, 9, 32, 43, 56, 59–61]. To examine if the NDR is maintained in Drosophila in the absence of binding proteins, we reconstituted chromatin *in vitro* using purified genomic DNA from *Drosophila* S2 cells and purified histone octamers from chicken erythrocytes [30]. We generated, sequenced and analyzed *in vitro* nucleosome maps as previously described, capturing over 25 million unique read pairs [36, 44]. Overall, nucleosome positioning around TSSs is much weaker in the *in vitro* reconstituted chromatin than in the *in vivo* chromatin (Fig 2A–2D), which suggests that much of the nucleosome organization around promoters requires dynamic regulation by DNA binding proteins. However, the *in vitro* map does show some positioning of the +1 nucleosomes in highly expressed genes suggesting that the DNA sequence plays a role in positioning this nucleosome (Fig 2B, arrowhead). In addition, the *in vitro* data show evidence of a positioned nucleosome over the nucleosome-depleted region at the TSS in highly expressed genes (Fig 2B, arrow). This suggests that preferential positioning of a nucleosome in the NDR is overridden in some actively transcribed genes. Higher expression levels strongly correlate with a more defined NDR, stronger positioning of the +1 nucleosome and more uniform nucleosome organization demonstrating that chromatin structure can reflect gene regulation. Taken together, our data indicates that while a large part of the global nucleosome organization in each cell line results from sequence-directed nucleosome positioning preferences, the positioning of nucleosomes near genes is strongly correlated with gene expression.

### Nucleosomal occupancy in different functional regions of the genome is similar between all cell lines

**Fig 3:**
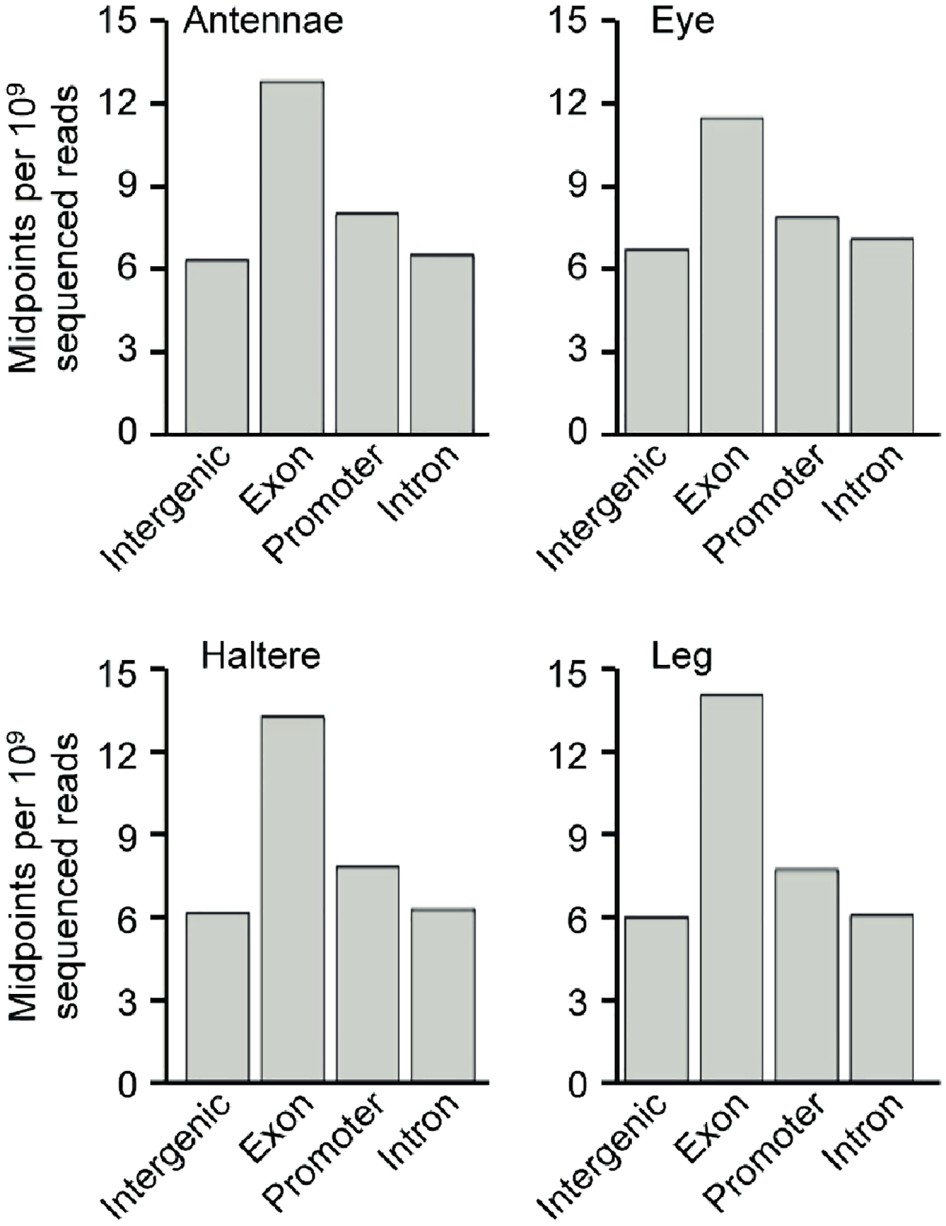
The density of nucleosomal reads in distinct genomic regions is alike for each cell type. For each of the *Drosophila* cell lines, the midpoint density of nucleosomal reads was counted and categorized by genomic region: promoters, exons, introns, and intergenic regions. Promoter regions were defined as 500 bp upstream and 500 bp downstream of the transcription start site. The higher density of nucleosomes in exons may result from higher GC content relative to intergenic, promoter or intron regions.

We next asked if cell line nucleosome occupancy agrees between different genomic regions that are important in gene regulation. Here we consider intergenic, intronic and exonic genomic regions. Regions were categorized using FlyBase gene annotations, with regions within 500 bp of an annotated transcription start sites (TSS) being defined as promoters. The number of nucleosome midpoints within each region were counted and normalized against the total number of sequenced aligned reads from each experiment to determine nucleosome enrichment in that region (Fig 3). Nucleosome occupancy was much higher in exons than in introns in all cell lines. This agrees with nucleosomal DNA sequence preferences, since exon DNA sequences generally have a higher G+C content than intron DNA sequences and therefore are less likely to contain the nucleosome-disfavoring poly-A kmers [18, 20, 32, 56]. Overall, the relative abundance of nucleosomes in each region agrees with previous studies [62, 63] and demonstrates that global nucleosome organization is not markedly different between cell types, and therefore that small-scale changes are likely to be important for cell type specification.

### Specific sequence motifs have differential nucleosome occupancy in cell lines and *in vitro* reconstituted chromatin

**Fig 4:**
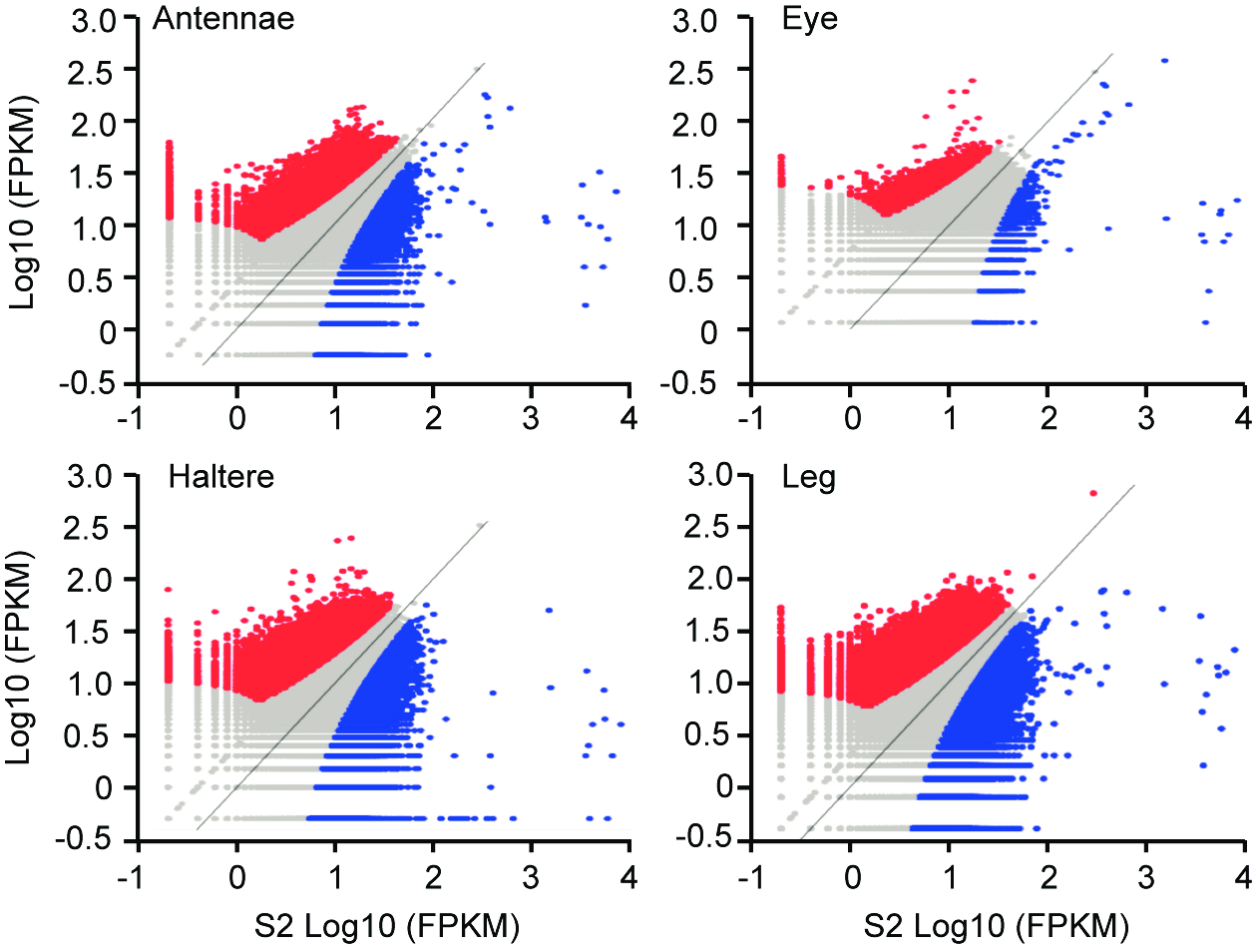
Comparison of nucleosome coverage between 200 bp regions in each cell line. The fragments per kilobase per million mapped reads (FPKM) for non-overlapping 200 bp windows was calculated along the genome in each cell line. This was then plotted against the FPKM obtained for a reference cell line (S2 cells) Each point represents a 200 bp region of the genome. Regions where the FPKM match closely are found within the grey area along the diagonal line. Areas with maximal change in the examined cell line and with the reference cell line are found in the red and blue regions respectively. Each colored region represents a large (> 2 fold difference in FPKM) and significant (false discovery rate FDR < 0.01) difference in the number of counts between S2 and the cell line it is being compared to. Pearson correlation values for antennae: R = 0.697, eye: R = 0.735, haltere: R = 0.646, leg: R = 0.672, p < 2.2e-16.

Given that the inherent nucleosome organization is broadly similar in each cell line, we hypothesized that changes in chromatin structure associated with cell-type specific expression occur locally, within smaller regulatory regions. To investigate this possibility, we divided the genome into non-overlapping 200 bp regions and compared the nucleosome coverage of each base pair in each cell line to the coverage in the S2 cells. S2 cells are derived from embryonic hemocyte (macrophage-like) cells, and thus provide a comparison for the four imaginal disc cell lines derived from later stage larval epithelial tissue. Although nucleosome occupancy within the different cell lines is generally similar to that of the S2 cell line, a subset of regions are markedly different (Fig 4), with many regions differing between 2 and 10-fold, and some regions differing by as much as 100-fold. These regions differ in that some are enriched for nucleosomes and some are depleted compared to the same region in the S2 cells.

To further resolve small differences between the four tissue-specific cell lines, we examined the nucleosomal occupancy over short kmers for each cell type. We used 7 bp kmers (i.e. heptamers) for analysis, reasoning that some differences between cell lines are likely to be at cell-type specific TF binding sites (TFBSs). TFBSs are short degenerate sequences, generally 7–11 bp, that occur throughout the genome [64, 65]. The context of any TFBS is important for regulatory function; TFBSs found within sequences that are highly favorable to nucleosome binding may be inaccessible to TFs and therefore may not be active [39, 66]. While many TFBSs have been annotated, we wanted to examine all possible 7 bp kmers to undertake an unbiased investigation in to whether specific kmers might correlate with differential nucleosome occupancies in differentiated cell lines. We expected to identify heptamers corresponding to the more than 700 TFBS motifs that have been discovered and annotated in the *Drosophila* genome [33], but we also hoped to identify previously unannotated sequences that are correlated with differential nucleosome occupancy.

We examined the extent to which nucleosome occupancy differs over all possible 16,384 heptamers between cell lines by dividing the genome into 200 bp regions, and calculating the average nucleosomal read depth in each 200 bp region, surrounding every occurrence of a 7-mer. The genome-wide average rate for each heptamer was calculated and normalized to the total number of nucleosomal reads sequenced in each lineage. We performed pairwise comparisons of the rate for each heptamer across the following cell lines and conditions; the four imaginal disc cell lines (antenna, eye, haltere and eye), the mean of all 4 cell lines, and the *in vitro* reconstituted chromatin. In total we performed 15 pairwise comparisons, and for each comparison, we considered the 20 kmers with the largest absolute residuals from the regression line to be “outliers”. In total, there were 91 unique outlier heptamers that had the greatest differences in at least one pairwise comparison (Table 2).

**Table 2:** Summary of heptamers with differential nucleosome occupancy among cell lines and in vitro chromatin.

Heptamer	Residuals from best fit line				
	antennae	eye	haltere	leg	in vitro
**AAAAAAA**	0.018	0.121	-0.035	-0.057	-0.049
**AAAAAAC**	0.009	0.056	-0.028	-0.017	-0.041
**AAAAAAG**	0.006	0.052	-0.028	-0.014	-0.01
**AAAAAAT**	0.012	0.073	-0.023	-0.03	-0.036
**AAAAATT**	0.007	0.065	-0.028	-0.015	0.001
**AAAATTT**	0.008	0.058	-0.032	-0.006	0.018
**AAATATA**	0.021	0.017	0.017	-0.032	-0.095
**AACAACA**	0.001	-0.004	0.026	-0.023	-0.183
**AACAGCA**	0.003	-0.006	0.033	-0.022	-0.209
**AAGGGGG**	0.004	0.041	-0.026	-0.002	0.189
**AATAATA**	0.021	0.03	0.013	-0.041	-0.06
**AATATAT**	0.026	0.011	0.024	-0.038	-0.085
**ACAACGA**	-0.005	-0.008	0.037	-0.023	-0.065
**ACAGCAG**	0.004	0.001	0.030	-0.021	-0.187
**ACATATA**	0.01	0.007	0.033	-0.042	-0.08
**ACCAACG**	-0.009	-0.007	0.043	-0.023	-0.066
**ACCCCCC**	0.008	0.082	-0.037	-0.015	0.277
**ACGCGCG**	-0.002	0.024	0.003	-0.008	0.004
**ACGTATA**	-0.009	-0.007	0.030	-0.018	-0.05
**ACGTTGG**	-0.008	-0.009	0.032	-0.015	-0.029
**AGCAACA**	0.002	-0.005	0.040	-0.027	-0.225
**AGCAGCA**	0.009	0.003	0.040	-0.028	-0.25
**AGCGCGC**	-0.013	0.072	-0.005	-0.022	-0.061
**AGGGGGG**	0.009	0.081	-0.036	-0.017	0.279
**ATAATAA**	0.016	0.034	0.013	-0.042	-0.05
**ATAATAT**	0.023	0.01	0.024	-0.036	-0.07
**ATACATA**	0.005	0.007	0.030	-0.036	-0.109
**ATACGCC**	-0.019	-0.004	0.023	0.000	-0.004
**ATATAAT**	0.018	0.009	0.027	-0.035	-0.07
**ATATACA**	0.009	0.007	0.030	-0.039	-0.097
**ATATATA**	0.051	0.022	0.045	-0.077	-0.071
**ATATATG**	0.008	0.003	0.030	-0.037	-0.069
**ATATGTA**	0.007	0.004	0.030	-0.035	-0.107
**ATCACCG**	0.015	0.08	0.018	-0.063	-0.033
**ATCGTTG**	-0.011	-0.023	0.045	-0.017	-0.047
**ATGTATA**	0.014	0.002	0.031	-0.038	-0.101
**ATTATTA**	0.018	0.029	0.012	-0.039	-0.045
**CAAAAAA**	0.008	0.059	-0.028	-0.019	-0.026
**CAACAAC**	0.000	-0.01	0.038	-0.025	-0.173
**CAACAGC**	0.004	-0.006	0.037	-0.023	-0.198
**CAACGAC**	-0.005	-0.003	0.043	-0.027	-0.037
**CACCCCC**	0.003	0.059	-0.021	-0.01	0.217
**CAGCAAC**	0.005	-0.007	0.043	-0.026	-0.228
**CAGCAGC**	0.011	0.009	0.037	-0.029	-0.234
**CATACGC**	-0.017	0.002	0.024	-0.006	-0.034
**CCAACGA**	-0.017	-0.018	0.060	-0.023	-0.006
**CCACCCC**	0.002	0.047	-0.017	-0.007	0.207
**CCCACCC**	0.002	0.031	-0.013	-0.002	0.19
**CCCCCCA**	0.004	0.056	-0.028	-0.007	0.211
**CCCCCCC**	0.019	0.177	-0.062	-0.048	0.513
**CCCCCCG**	0.005	0.054	-0.027	-0.005	0.225
**CCCCCGC**	0.004	0.063	-0.021	-0.012	0.179
**CCCCGCC**	0.005	0.054	-0.015	-0.013	0.186
**CCCCTCC**	0.005	0.04	-0.019	-0.004	0.196
**CCCTCCC**	0.005	0.046	-0.022	-0.006	0.196
**CCGCCGC**	0.029	0.062	-0.009	-0.037	0.073
**CCTCCCC**	0.006	0.049	-0.023	-0.007	0.203
**CGCATAC**	-0.017	0.003	0.023	-0.008	-0.05
**CGCCGCC**	0.026	0.056	-0.008	-0.033	0.081
**CGCGCCC**	0.002	0.066	-0.013	-0.019	0.06
**CGCGCCG**	-0.001	0.063	-0.008	-0.02	0.011
**CGCGCGA**	-0.003	0.062	-0.015	-0.015	-0.033
**CGCGCGC**	-0.011	0.108	-0.016	-0.034	-0.077
**CGCGCTA**	-0.017	0.044	-0.005	-0.005	-0.039
**CGCTCTC**	-0.006	0.068	-0.015	-0.019	-0.051
**CGGCCGC**	0.013	0.061	-0.005	-0.03	0.057
**CGGCGCC**	0.017	0.059	-0.006	-0.029	0.044
**CGGCGGC**	0.026	0.056	-0.005	-0.035	0.058
**CGTATAC**	-0.021	0.00	0.033	-0.012	-0.038
**CGTATGC**	-0.016	0.004	0.023	-0.008	-0.042
**CGTTGGC**	-0.011	-0.003	0.040	-0.019	-0.002
**CTATATA**	0.009	-0.003	0.030	-0.034	-0.074
**CTGCTGC**	0.011	0.011	0.033	-0.028	-0.215
**CTGTTGC**	0.005	-0.001	0.034	-0.024	-0.191
**GAAAAAA**	0.004	0.066	-0.031	-0.017	0.006
**GATCACC**	0.022	0.023	0.016	-0.038	-0.066
**GCAGCAA**	0.00	-0.002	0.032	-0.02	-0.19
**GCCCCCC**	0.009	0.061	-0.024	-0.013	0.224
**GCGCGAA**	-0.01	0.046	-0.015	-0.005	-0.023
**GCGCGCA**	-0.012	0.064	-0.001	-0.022	-0.08
**GCGCGCC**	-0.002	0.073	-0.005	-0.026	-0.03
**GCGTATA**	-0.019	0.003	0.026	-0.01	-0.024
**GGGGGGA**	0.002	0.054	-0.030	-0.004	0.222
**GTATATA**	0.011	0.003	0.034	-0.04	-0.061
**TAAAAAA**	0.017	0.059	-0.018	-0.025	-0.067
**TAATATA**	0.031	0.008	0.034	-0.043	-0.099
**TACTATA**	0.004	0.004	0.033	-0.036	-0.097
**TAGTATA**	0.009	0.00	0.032	-0.033	-0.095
**TATAATA**	0.026	0.01	0.034	-0.042	-0.078
**TATATAA**	0.025	0.004	0.032	-0.039	-0.087
**TTCGAAA**	0.002	0.016	-0.038	0.022	0.019

Shown in alphabetical order are the 91 unique differentially occupied heptamers that were among the top 20 outliers in any of the pairwise comparison of individual cell lines and in vitro chromatin. Residual values are shown for the comparison of specific cell lines to the mean rate from all cell lines (antennae, eye, haltere, and leg), and for the combined rate in all lines to the rate in *in vitro* reconstituted chromatin (*in vitro*).

**Fig 5:**
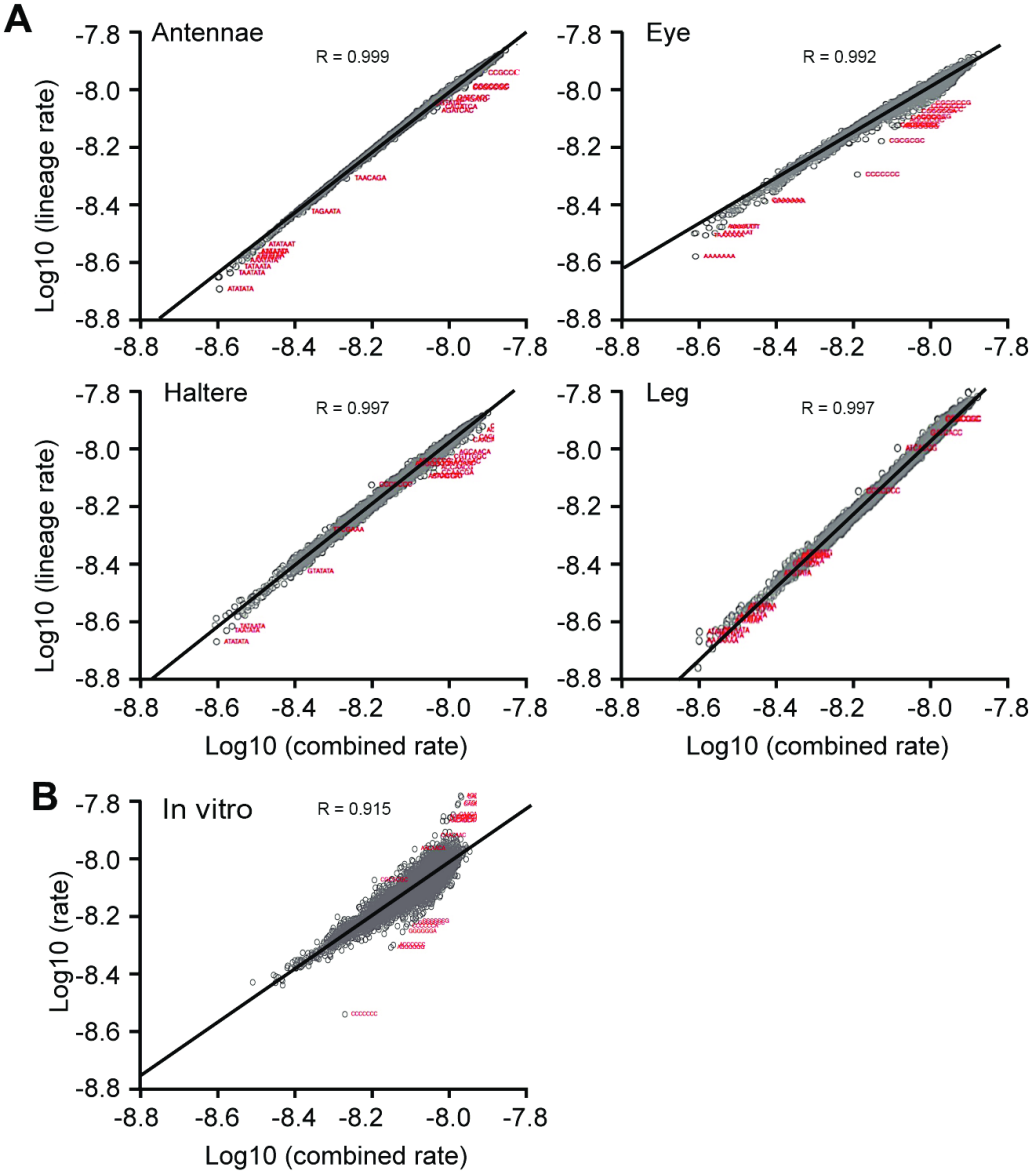
Nucleosome coverage of specific heptamers is not identical in all cell lines. **(**A) Comparison of the rate of nucleosome coverage for each heptamer in each cell line to the rate of that heptamer in all four of the cell lines combined. Nucleosome coverage over most heptamers was comparable in all cell lines (R>0.99 for all comparisons). However, some heptamers were over-represented or under-represented in specific cell lines and thus appeared as dots off the main line. The 20 heptamers with the largest absolute residual values were considered outliers and are highlighted in red. (B) The combined rate of each heptamer from the cell lines compared to the rate of that heptamer in *in vitro* reconstituted chromatin. Outliers marked in red as described in part A.

In general, nucleosome occupancy over heptamers was highly correlated across cell lines as seen in Fig 5A (R values > 0.99). Furthermore, this correlation was maintained even when compared to the *in vitro* reconstituted chromatin (Fig 5B) (R value = 0.91), demonstrating that genomic sequence plays a key role in global nucleosome positioning, directly through DNA-histone interactions. However, multiple outliers were observed that were either more- or less-occupied by nucleosomes relative to their coverage in other cell lines (Table 2, Fig 5, outliers annotated with red text). These findings suggest that while nucleosome placement is generally guided by thermodynamics and the underlying DNA sequence, there are differences in nucleosome occupancy for specific kmers between datasets that are likely caused by energetically driven processes.

**Fig 6:**
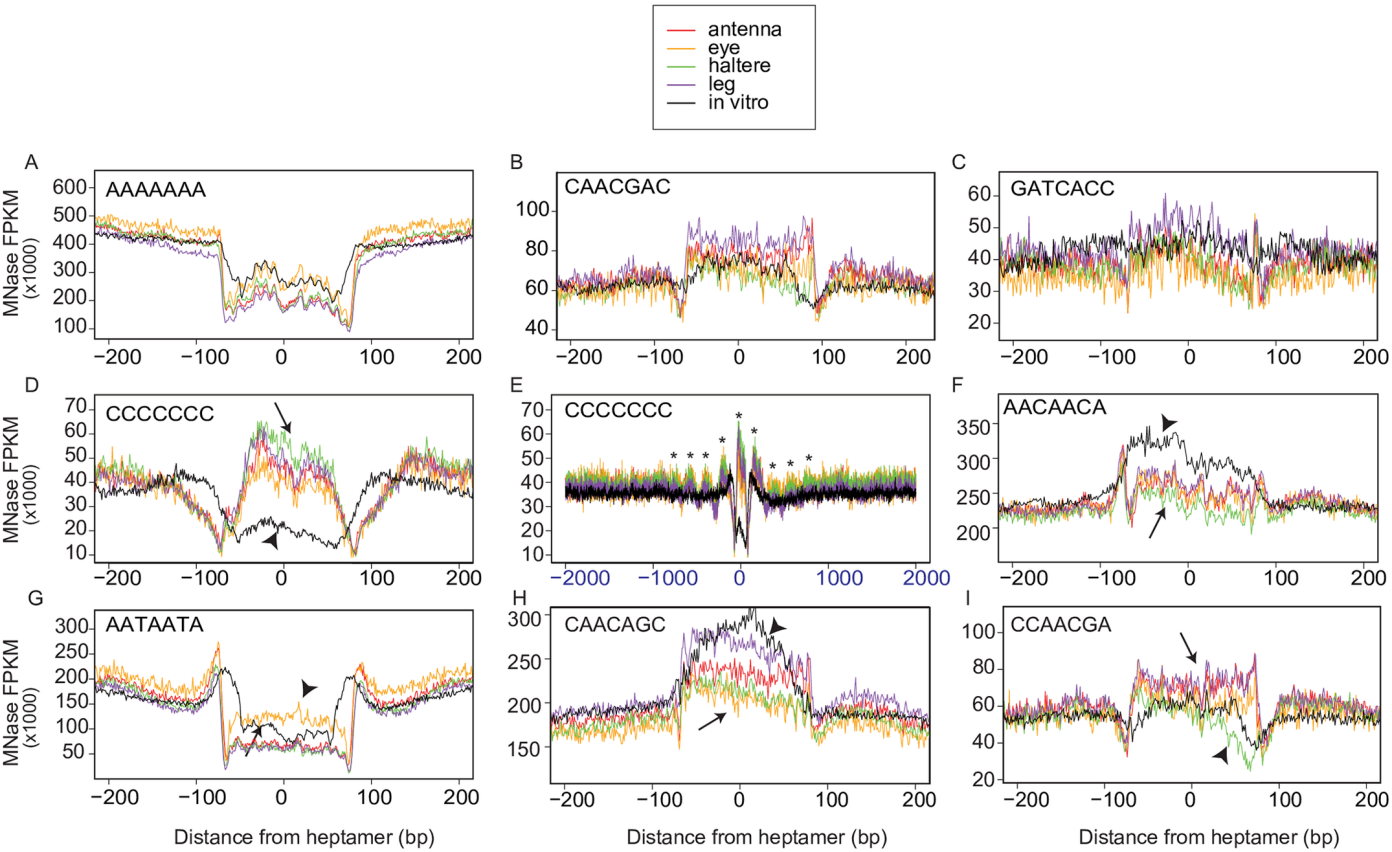
Specific heptamers show different patterns of nucleosome occupancy between cell lines and also between cell lines and *in vitro* chromatin. Mapped nucleosome midpoints centered over heptamer sequences revealed several different patterns of nucleosome positioning. (A) AT-rich heptamers that exclude nucleosomes both *in vivo* and *in vitro* showed “depleted” patterns centered on the sequence, such as the poly-A kmer AAAAAAA. (B) “Covered” motifs such as CAACGAC showed increased occupancy in all datasets. (C) “Noisy” motifs such as GATCACC showed no discernible pattern of nucleosome occupancy centered on the heptamer. (D) The homopolymeric motif CCCCCCC was depleted of nucleosomes in the *in vitro* dataset only (arrow marks *in vivo* occupancies, arrowhead indicates *in vitro* chromatin). (E) Long range ordering could be seen in the +/- 2000bp surrounding poly-C heptamers. Visibly phased nucleosomes marked with asterisks. (F) In contrast to the CCCCCC motif in (D), the AACAACA motif was more covered in the *in vitro* dataset than in the cell lines (arrowhead marks *in vitro* chromatin, arrow marks *in vivo*). (G-H) Some motifs showed cell line-specific occupancy of heptamers. Whereas AATAATA was more covered in the eye cell line (yellow line marked by arrowhead in G) than other cell lines or *in vitro* chromatin, CAACAGC was more covered in the leg cell line (H, arrowhead and purple line vs arrow other lines indicated by arrow), and CGAACGA was less covered in the haltere cell line (I, green line indicated by arrowhead vs other lines indicated by arrow).

To determine if any of the differentially occupied heptamer sequences correlated with positioned nucleosomes, we visualized the nucleosome occupancy surrounding specific heptamers by aggregating nucleosome midpoints across occurrences of the heptamer and plotting the mean midpoint density in 400 bp regions centered on the heptamer. Interestingly, nucleosome occupancy surrounding the heptamers varied considerably around different heptamers. For some heptamers, there was a visible reduction or increase in nucleosomal occupancy surrounding the heptamer in all cell lines and in the *in vitro* chromatin (e.g. Fig 6A and 6B, respectively). For other heptamers, nucleosome occupancy surrounding the heptamer site showed no discernable pattern (e.g Fig 6C). In multiple cases, we observed differential nucleosome coverage between cell lines and the *in vitro* chromatin, with either the cell line or the *in vitro* chromatin having greater nucleosome coverage (Fig 6D and 6F arrows indicate occupancy in cell lines, arrowheads indicate occupancy *in vitro* chromatin). Notably, in some cases, the region of differential nucleosome occupancy was tightly centered on the heptamer sequences but phasing of nucleosomes extended to broader genomic regions (e.g. Fig 6E, asterisks indicate periodic peaks). We also identified several cases where nucleosome occupancy around specific heptamers differed in only one of the cell lines (Fig 6F–6H). For example, the heptamer AATAATA has reduced nucleosome occupancy in the leg, antenna and haltere lines (Fig 6G, arrow), but is distinctly more occupied in the eye cell line (Fig 6G yellow line indicated by arrowhead). Conversely, the CAACAGC heptamer is slightly over-occupied in eye, haltere, and antennal cell lines (Fig 6H arrow), but is visibly more occupied in the leg cell line (Fig 6H, purple line indicated arrowhead). Reduced occupancy in the haltere cell line (Fig 6I green line indicated by arrowhead) is observed over CCAACGA motifs compared to the other cell lines and *in vitro* (Fig 6I arrow). Together, these results demonstrate that that, over heptamers, nucleosome organization is driven to a large extent by DNA sequence, but there are nonetheless clear differences between *in vivo* and in vitro nucleosome organization, as well as cell line-specific differences.

### Some differentially occupied heptamer sequences correspond to annotated transcription factor binding sites

We next asked whether any of the heptamers with differential occupancy between the cell lines match known regulatory sequences. Using the Tomtom motif comparison tool of the MEME suite of tools (www.meme-suite.org), we compared the differential heptamers to those in databases of known *Drosophila* transcription factor binding sites. Of the 91 differentially occupied heptamers identified, 49 matched one or more known *Drosophila* TFBS consensus sequences (Table 3) The transcription factors for these TFBSs have a wide array of biological functions, but several stand out as being important in cell type specification. The poly (dC:dG) heptamers CGCCGCC and CCCCCCC match the predicted binding sites of Buttonhead (Btd) and Brinker (Brk), two transcription factors involved in imaginal disc antennal and wing morphogenesis respectively. Notably, both of these heptamers are associated with regions of differential nucleosome occupancy (Fig 6D–6F). Conversely, another transcriptional factor involved in imaginal disc development, Rotund (Rn), binds to homopolymeric A/T sequences [67], which also shows differential nucleosome occupancy (Fig 6A). Bric à brac 1 (Bab1**)** is a TF that is needed in appendage formation [68]. Interestingly, the three cell lines derived from tissues that normally form appendages (antennal, leg, and haltere) all have open chromatin structure over the AATAATA motif that matches the Bab1 binding sequence, whereas this site shows higher nucleosomal occupancy in the eye cell lineage (Fig 6F). These results suggest that some of the 42 heptamers that do not correspond to known TFBS might in fact interact with binding factors to influence nucleosome positioning and/or gene expression.

**Table 3:** 49 heptamers match the consensus binding sequences of transcription factors.

TFs	Matching Query Heptamers for TFBSs							
**CG12605**	AAAAAAA	CTGTTGC						
**Hb**	AAAAAAA	AAAAAAC	CAAAAAA	TAAAAAA				
**Jigr1**	AAAAAAA	TAAAAAA						
**Jim**	AAAAAAA	CAAAAAA	AAAAAAC					
**Rn**	AAAAAAA	AAAAAAC	CAAAAAA					
**Sqz**	AAAAAAA	CAAAAAA						
**Mirr**	AAAAAAC							
**Dati**	AAAAAAT	AAAAAAA						
**CG4360**	AACAACA	CAACAAC						
**Bteb2**	AAGGGGG	ATACGCC	CGCCGCC	CGCGCCC	ACCCCCC	CCCCCCC	GCCCCCC	
**Bab1**	AATAATA							
**Bin**	ACAACGA							
**Cf2-II**	ACATATA	ATATATA	ATATATG	ATATGTA	GCGTATA	TACTATA	TAGTATA	
**Ci**	ACCCCCC	CCCACCC	CCCCCCA					
**CG11504**	AGCAACA	CAGCAAC	CCCACCC	CACCCCC				
**Top2**	ATATGTA	ATACATA						
**Aef1**	CAACAAC	AACAACA	AAAAAAA					
**HLH4C**	CAGCAAC							
**Dar1**	CCCACCC	CCCCCCA	GCCCCCC	CCGCCGC	CCCCCCG	CCCCCGC	CCCCTCC	
	CCCACCC	CACCCCC						
**Klu**	CCCACCC	CCCCCCA	CCCCCGC	CACCCCC	CCACCCC			
**Ttk**	CCCACCC	CACCCCC	CCACCCC					
**Ara**	CCCCCCC	AAAAAAC						
**Btd**	CCCCCCC	CCTCCCC	GCCCCCC	CCGCCGC				
**CG7368**	CCCCCCC	CCACCCC	GGGGGGA	CCCTCCC	CCCACCC	CCCCCCA	CCCCTCC	CACCCCC
**L(3)neo38**	CCCCCCC	GGGGGGA	AGGGGGG	ACCCCCC	CCCACCC	CCCCCCA	CACCCCC	CCACCCC
	CCCCCGC	CACCCCC						
**CG3065**	CCCCGCC	CGCGCCC	GCCCCCC	CACCCCC	CCACCCC			
**CG42741**	CCCCGCC							
**Crol**	CCCCGCC	CCCCCCC	GGGGGGA	AGGGGGG	CCCTCCC	CCTCCCC	ACCCCCC	CACCCCC
**Hnf4**	CCCCGCC							
**Lmd**	CCCCGCC	CCCCCCC	ACCCCCC	CCCCCCA	GCCCCCC	CCCCCCG	CCCCCGC	CACCCCC
**Luna**	CCCCGCC							
**Opa**	CCCCGCC	CCCCCCC	GGGGGGA	ACCCCCC	CCCCCCA	GCCCCCC	CCGCCGC	CCCCCCG
**Sp1**	CCCCGCC	GCCCCCC						
**Spps**	CCCCGCC	CCGCCGC	CGCCGCC					
**Sr**	CCCCGCC	ACCCCCC	CCCACCC	CCCCCGC				
**Sug**	CCCCGCC	CCCCCCC	ACCCCCC	CCCCCCA	GCCCCCC	CCCCCCG	CCCCCGC	
**Pad**	CCCCTCC							
**Med**	CCGCCGC							
**Hkb**	CGCGCCC	CACCCCC						
**E(spl)mbealpa-HLH**	CGCGCGC	GCGCGCC						
**H**	CGCGCGC	GCGCGCC	CGCGCCG					
**Trl**	CGCTCTC							
**Adf1**	CGGCCGC							
**Lola**	CGGCCGC	CCCCCCC	GGGGGGA	AGGGGGG	CCCCCCA	GCCCCCC	CCCCCCG	ACCCCCC
**Brk**	CGGCGCC							
**Mad**	CGGCGCC	CGCCGCC	CGCGCCG	CGGCGGC				
**Scrt**	CTGTTGC							
**Fru**	GAAAAAA							
**CG8319**	GATCACC							
**CG3838**	GCGTATA	AGCAACA						
**Shn**	GGGGGGA							

## Discussion

Previous studies have revealed canonical patterns of nucleosome organization in the genomes of many different organisms [21, 22, 54–56, 69]. However, few studies have examined nucleosome organization in the context of differences between distinct cell lines. Our goal in this study was to provide analysis of nucleosome positioning in five cultured cell lines from a model organism, *Drosophila melaogaster*, and determine if there is evidence that short sequences can influence nucleosome positioning and occupancy. Such evidence would serve as a basis for future causal investigations into the relationship between nucleosome positioning and cell-type specification, and for possibly identifying factors that bind these short sequences.

The presented results show that while underlying sequence does play a role in nucleosome occupancy, there are notable differences in nucleosome occupancy between the cell lines examined and *in vitro* reconstituted chromatin. We also identified cell type-specific differences that are distinct from the DNA sequences expected to favor or disfavor nucleosome positioning. These results are in line with other studies showing differential nucleosome occupancy during cell-type regulation [11, 41], and suggest that changes in nucleosome positioning could be involved in cell fate specification and maintenance. Importantly, our studies extend previous results by identifying 91 heptamers that show cell type-specific nucleosome occupancy. In some cases, strong nucleosome-positioning patterns extend in excess of 1,000 base pairs into the region surrounding the heptamer. While 49 of these heptamers correspond to binding sites of known transcription factors, 42 heptamers do not correspond to known binding factors. We speculate that these novel heptamers identify binding sites for transcription or chromatin remodeling factors that have important roles in establishing specific cell fate in the studied lines.

The possibility that these 42 heptamers could be functional binding sites for transcription factors or chromatin remodeling factors is supported by our finding that 49 of the differentially occupied heptamers corresponded to binding sites for known transcription factors. A particularly notable example is Bab1 (*bric á brac*), a transcription factor required for appendage development [68]. The heptamer that matches the Bab1 binding sequence, AATAATA, has an open chromatin motif in the three cell lines derived from tissues that normally form appendages (antennal, leg, and haltere), but this heptamer shows higher nucleosome occupancy in the eye-derived cell line. Further studies will be necessary to determine if any of the 42 novel heptamers in fact bind trans-acting factors, and whether they causally affect nucleosome positioning. However, if binding of factors to these sites does influence nucleosome positioning, as detailed below, we would also expect that the corresponding heptamer could influence gene expression.

What is the relationship between nucleosome organization and gene expression in these cell type-specific cell lines? Our results show that, as in embryos and other organisms, highly expressed genes in these cell lines show specific organization of nucleosome with an NDR at the TSS, and phased nucleosomes distal to the TSS [27, 55, 56, 58, 59, 70] As has been observed in other species, genes with low expression did not have an organized nucleosome pattern [27, 56]. This correlation, and work in multiple organisms [43, 51, 56, 58, 59], suggests that binding of a transcription factor or chromatin remodeling factors to a heptamer sequence could alter nucleosome positioning, which could alter gene transcription, and thus alter cell fate specification. Alternatively, since the canonical nucleosome occupancy pattern observed in highly expressed genes likely creates a chromatin structure best poised for RNA polymerase or TF binding [49, 50, 56, 71], an open chromatin environment created by upstream signaling events could allow a specific TF to bind and thus contribute to cell fate specification or maintenance. Further work is needed to establish whether binding of factors to heptamers alters nucleosome organization or whether altered nucleosome organization allows access and binding of regulating factors.

In summary, our data demonstrates that a large part of the *in vivo* global nucleosome organization in each cell line results from nucleosome-positioning preferences, favorable and unfavorable, encoded in the DNA. Genomic encoding of nucleosome preference is an integral component of gene regulation. However, overriding the effect of the underlying sequence is cell-type specific nucleosome organization that is mediated by other factors such as TFs and chromatin remodelers [11, 31, 41]. Our data contribute useful datasets of genome-wide nucleosome positioning in distinct *Drosophila* cell lines and identify heptamers that are differentially occupied in different cell lines. While 49 of these heptamers match binding sites of known TFs, 42 have no current match, and thus define possible binding sites for novel cell fate specification factors. Together, these data provide tools for examining the effect of sequence and functional relationships between transcription factor activity, nucleosome location in gene regulation and cell fate specification.

## Supporting information

S1 Fig Mononucleosomal fragments from all cell lines show similar fragment size distribution. The distribution of the sequenced mononucleosomal DNA fragment lengths is very similar across all cell lines.
(PDF)

S2 Fig Nucleosome profiles along arbitrary *Drosophila* genomic regions maintain features between cell lines. MNase midpoint density profiles, smoothed using a 30 bp sliding window, along randomly chosen genomic regions demonstrate that the nucleosome arrays from each cell line (top four tracks) correspond well with one another. The in vitro nucleosome arrays from this study (green, bottom tracks) correspond least well with nucleosome arrays generated from cells but maintain similar spacing and many of the strong and intermediate peaks. Example peaks that are similar between the cell lines and *in vitro* reconstituted chromatin are indicated with black boxes, while example peaks that are strong in all cell lines but reduced in in vitro chromatin, or vice versa, are shown in red boxes and marked with an asterisk.
(PDF)

## References

[1] Luger K, Mader A, Sargent DF, Richmond TJ. The atomic structure of the nucleosome core particle. Journal of biomolecular structure & dynamics. 2000;17 Suppl 1:185–8. Epub 2000/01/01. 10.1080/07391102.2000.10506619 . 22607422

[2] Kaplan N, Moore IK, Fondufe-Mittendorf Y, Gossett AJ, Tillo D, Field Y, et al The DNA-encoded nucleosome organization of a eukaryotic genome. Nature. 2009;458(7236):362–6. Epub 2008/12/19. 10.1038/nature07667 . 19092803 PMC2658732

[3] Lowary PT, Widom J. New DNA sequence rules for high affinity binding to histone octamer and sequence-directed nucleosome positioning. Journal of molecular biology. 1998;276(1):19–42. Epub 1998/03/26. 10.1006/jmbi.1997.1494 . 9514715

[4] Walter PP, Owen-Hughes TA, Cote J, Workman JL. Stimulation of transcription factor binding and histone displacement by nucleosome assembly protein 1 and nucleoplasmin requires disruption of the histone octamer. Molecular and cellular biology. 1995;15(11):6178–87. Epub 1995/11/01. . 7565770 10.1128/mcb.15.11.6178 PMC230869

[5] Straka C, Horz W. A functional role for nucleosomes in the repression of a yeast promoter. EMBO J. 1991;10(2):361–8. . 1899374 10.1002/j.1460-2075.1991.tb07957.x PMC452655

[6] Zhou J, Fan JY, Rangasamy D, Tremethick DJ. The nucleosome surface regulates chromatin compaction and couples it with transcriptional repression. Nat Struct Mol Biol. 2007;14(11):1070–6. 10.1038/nsmb1323 . 17965724

[7] Grunstein MHaM. Nucleosome loss activates yeast downstream promoters in vivo Cell. 1988;55:1137–45. 2849508 10.1016/0092-8674(88)90258-9

[8] Raveh-Sadka T, Levo M, Segal E. Incorporating nucleosomes into thermodynamic models of transcription regulation. Genome research. 2009;19(8):1480–96. Epub 2009/05/20. 10.1101/gr.088260.108 . 19451592 PMC2720181

[9] Schones DE, Cui K, Cuddapah S, Roh TY, Barski A, Wang Z, et al Dynamic regulation of nucleosome positioning in the human genome. Cell. 2008;132(5):887–98. Epub 2008/03/11. 10.1016/j.cell.2008.02.022 . 18329373 PMC10894452

[10] Hellauer K, Sirard E, Turcotte B. Decreased expression of specific genes in yeast cells lacking histone H1. The Journal of biological chemistry. 2001;276(17):13587–92. Epub 2001/03/30. 10.1074/jbc.M011196200 . 11278859

[11] Yigit E, Bischof JM, Zhang Z, Ott CJ, Kerschner JL, Leir SH, et al Nucleosome mapping across the CFTR locus identifies novel regulatory factors. Nucleic acids research. 2013;41(5):2857–68. Epub 2013/01/18. 10.1093/nar/gks1462 . 23325854 PMC3597660

[12] Jiang C, Pugh BF. Nucleosome positioning and gene regulation: advances through genomics. Nature reviews Genetics. 2009;10(3):161–72. Epub 2009/02/11. 10.1038/nrg2522 . 19204718 PMC4860946

[13] Shivaswamy S, Bhinge A, Zhao Y, Jones S, Hirst M, Iyer VR. Dynamic remodeling of individual nucleosomes across a eukaryotic genome in response to transcriptional perturbation. PLoS biology. 2008;6(3):e65 Epub 2008/03/21. 10.1371/journal.pbio.0060065 . 18351804 PMC2267817

[14] Li B, Carey M, Workman JL. The role of chromatin during transcription. Cell. 2007;128(4):707–19. Epub 2007/02/27. 10.1016/j.cell.2007.01.015 . 17320508

[15] Gracey LE, Chen ZY, Maniar JM, Valouev A, Sidow A, Kay MA, et al An in vitro-identified high-affinity nucleosome-positioning signal is capable of transiently positioning a nucleosome in vivo. Epigenetics & chromatin. 2010;3(1):13 Epub 2010/07/03. 10.1186/1756-8935-3-13 . 20594331 PMC2915997

[16] Corona DF, Siriaco G, Armstrong JA, Snarskaya N, McClymont SA, Scott MP, et al ISWI regulates higher-order chromatin structure and histone H1 assembly in vivo. PLoS biology. 2007;5(9):e232 Epub 2007/09/01. 10.1371/journal.pbio.0050232 . 17760505 PMC1951781

[17] Yigit E, Zhang Q, Xi L, Grilley D, Widom J, Wang JP, et al High-resolution nucleosome mapping of targeted regions using BAC-based enrichment. Nucleic Acids Res. 2013;41(7):e87 10.1093/nar/gkt081 . 23413004 PMC3627574

[18] Moyle-Heyrman G, Zaichuk T, Xi L, Zhang Q, Uhlenbeck OC, Holmgren R, et al Chemical map of Schizosaccharomyces pombe reveals species-specific features in nucleosome positioning. Proceedings of the National Academy of Sciences of the United States of America. 2013;110(50):20158–63. Epub 2013/11/28. 10.1073/pnas.1315809110 . 24277842 PMC3864286

[19] Nalabothula N, Xi L, Bhattacharyya S, Widom J, Wang JP, Reeve JN, et al Archaeal nucleosome positioning in vivo and in vitro is directed by primary sequence motifs. BMC genomics. 2013;14:391 Epub 2013/06/14. 10.1186/1471-2164-14-391 . 23758892 PMC3691661

[20] Brogaard K, Xi L, Wang JP, Widom J. A map of nucleosome positions in yeast at base-pair resolution. Nature. 2012;486(7404):496–501. Epub 2012/06/23. 10.1038/nature11142 . 22722846 PMC3786739

[21] Segal E, Widom J. What controls nucleosome positions? Trends in genetics: TIG. 2009;25(8):335–43. Epub 2009/07/15. 10.1016/j.tig.2009.06.002 . 19596482 PMC2810357

[22] Radman-Livaja M, Rando OJ. Nucleosome positioning: how is it established, and why does it matter? Dev Biol. 2010;339(2):258–66. Epub 2009/06/17. 10.1016/j.ydbio.2009.06.012 . 19527704 PMC2830277

[23] Luger K, Mader AW, Richmond RK, Sargent DF, Richmond TJ. Crystal structure of the nucleosome core particle at 2.8 A resolution. Nature. 1997;389(6648):251–60. Epub 1997/09/26. 10.1038/38444 . 9305837

[24] Rapoport AE, Frenkel ZM, Trifonov EN. Nucleosome positioning pattern derived from oligonucleotide compositions of genomic sequences. Journal of biomolecular structure & dynamics. 2011;28(4):567–74. Epub 2010/12/15. 10.1080/07391102.2011.10531243 . 21142224

[25] Ioshikhes I, Hosid S, Pugh BF. Variety of genomic DNA patterns for nucleosome positioning. Genome research. 2011;21(11):1863–71. Epub 2011/07/14. 10.1101/gr.116228.110 . 21750105 PMC3205571

[26] Tilgner H, Nikolaou C, Althammer S, Sammeth M, Beato M, Valcarcel J, et al Nucleosome positioning as a determinant of exon recognition. Nat Struct Mol Biol. 2009;16(9):996–1001. Epub 2009/08/18. 10.1038/nsmb.1658 . 19684599

[27] Lee W, Tillo D, Bray N, Morse RH, Davis RW, Hughes TR, et al A high-resolution atlas of nucleosome occupancy in yeast. Nature genetics. 2007;39(10):1235–44. Epub 2007/09/18. 10.1038/ng2117 . 17873876

[28] Suter B, Schnappauf G, Thoma F. Poly(dA.dT) sequences exist as rigid DNA structures in nucleosome-free yeast promoters in vivo. Nucleic Acids Res. 2000;28(21):4083–9. Epub 2000/11/01. . 11058103 10.1093/nar/28.21.4083 PMC113125

[29] Thastrom A, Lowary PT, Widlund HR, Cao H, Kubista M, Widom J. Sequence motifs and free energies of selected natural and non-natural nucleosome positioning DNA sequences. Journal of molecular biology. 1999;288(2):213–29. Epub 1999/05/18. 10.1006/jmbi.1999.2686 . 10329138

[30] Satchwell SC, Drew HR, Travers AA. Sequence periodicities in chicken nucleosome core DNA. J Mol Biol. 1986;191(4):659–75. . 3806678 10.1016/0022-2836(86)90452-3

[31] Prasad P, Lennartsson A, Ekwall K. The roles of SNF2/SWI2 nucleosome remodeling enzymes in blood cell differentiation and leukemia. Biomed Res Int. 2015;2015:347571 Epub 2015/03/20. 10.1155/2015/347571 . 25789315 PMC4348595

[32] Lorch Y, Maier-Davis B, Kornberg RD. Role of DNA sequence in chromatin remodeling and the formation of nucleosome-free regions. Genes & development. 2014;28(22):2492–7. Epub 2014/11/19. 10.1101/gad.250704.114 . 25403179 PMC4233242

[33] Cherbas L, Willingham A, Zhang D, Yang L, Zou Y, Eads BD, et al The transcriptional diversity of 25 Drosophila cell lines. Genome Res. 2011;21(2):301–14. 10.1101/gr.112961.110 . 21177962 PMC3032933

[34] Baum B, Cherbas L. Drosophila cell lines as model systems and as an experimental tool. Methods in molecular biology. 2008;420:391–424. Epub 2008/07/22. 10.1007/978-1-59745-583-1_25 . 18641962

[35] Sasse S, Neuert H, Klambt C. Differentiation of Drosophila glial cells. Wiley Interdiscip Rev Dev Biol. 2015;4(6):623–36. 10.1002/wdev.198 . 26178654

[36] Wolfstetter G, Holz A. The role of LamininB2 (LanB2) during mesoderm differentiation in Drosophila. Cell Mol Life Sci. 2012;69(2):267–82. 10.1007/s00018-011-0652-3 . 21387145 PMC11114671

[37] Minakhina S, Tan W, Steward R. JAK/STAT and the GATA factor Pannier control hemocyte maturation and differentiation in Drosophila. Dev Biol. 2011;352(2):308–16. 10.1016/j.ydbio.2011.01.035 . 21295568 PMC3065540

[38] Jasper H, Benes V, Atzberger A, Sauer S, Ansorge W, Bohmann D. A genomic switch at the transition from cell proliferation to terminal differentiation in the Drosophila eye. Dev Cell. 2002;3(4):511–21. . 12408803 10.1016/s1534-5807(02)00297-6

[39] Fu Y, Sinha M, Peterson CL, Weng Z. The insulator binding protein CTCF positions 20 nucleosomes around its binding sites across the human genome. PLoS genetics. 2008;4(7):e1000138 Epub 2008/07/26. 10.1371/journal.pgen.1000138 . 18654629 PMC2453330

[40] Breiling A, Bonte E, Ferrari S, Becker PB, Paro R. The Drosophila polycomb protein interacts with nucleosomal core particles In vitro via its repression domain. Molecular and cellular biology. 1999;19(12):8451–60. Epub 1999/11/24. . 10567570 10.1128/mcb.19.12.8451 PMC84949

[41] Sebeson A, Xi L, Zhang Q, Sigmund A, Wang JP, Widom J, et al Differential Nucleosome Occupancies across Oct4-Sox2 Binding Sites in Murine Embryonic Stem Cells. PloS one. 2015;10(5):e0127214 10.1371/journal.pone.0127214 . 25992972 PMC4436218

[42] Lei I, West J, Yan Z, Gao X, Fang P, Dennis JH, et al BAF250a Protein Regulates Nucleosome Occupancy and Histone Modifications in Priming Embryonic Stem Cell Differentiation. The Journal of biological chemistry. 2015;290(31):19343–52. Epub 2015/06/14. 10.1074/jbc.M115.637389 . 26070559 PMC4521052

[43] Valouev A, Ichikawa J, Tonthat T, Stuart J, Ranade S, Peckham H, et al A high-resolution, nucleosome position map of C. elegans reveals a lack of universal sequence-dictated positioning. Genome Res. 2008;18(7):1051–63. Epub 2008/05/15. 10.1101/gr.076463.108 . 18477713 PMC2493394

[44] Ui K, Ueda R, Miyake T. Cell lines from imaginal discs of Drosophila melanogaster. In Vitro Cell Dev Biol. 1987;23(10):707–11. . 3117765 10.1007/BF02620984

[45] Zhang Y, Malone JH, Powell SK, Periwal V, Spana E, Macalpine DM, et al Expression in aneuploid Drosophila S2 cells. PLoS biology. 2010;8(2):e1000320 Epub 2010/02/27. 10.1371/journal.pbio.1000320 . 20186269 PMC2826376

[46] Segal E, Fondufe-Mittendorf Y, Chen L, Thastrom A, Field Y, Moore IK, et al A genomic code for nucleosome positioning. Nature. 2006;442(7104):772–8. Epub 2006/07/25. 10.1038/nature04979 . 16862119 PMC2623244

[47] Thastrom A, Bingham LM, Widom J. Nucleosomal locations of dominant DNA sequence motifs for histone-DNA interactions and nucleosome positioning. Journal of molecular biology. 2004;338(4):695–709. Epub 2004/04/22. 10.1016/j.jmb.2004.03.032 . 15099738

[48] Chang GS, Noegel AA, Mavrich TN, Muller R, Tomsho L, Ward E, et al Unusual combinatorial involvement of poly-A/T tracts in organizing genes and chromatin in Dictyostelium. Genome Res. 2012;22(6):1098–106. Epub 2012/03/22. 10.1101/gr.131649.111 . 22434426 PMC3371697

[49] Anderson JD, Widom J. Poly(dA-dT) promoter elements increase the equilibrium accessibility of nucleosomal DNA target sites. Molecular and cellular biology. 2001;21(11):3830–9. Epub 2001/05/08. 10.1128/MCB.21.11.3830-3839.2001 . 11340174 PMC87046

[50] Yuan GC, Liu YJ, Dion MF, Slack MD, Wu LF, Altschuler SJ, et al Genome-scale identification of nucleosome positions in S. cerevisiae. Science. 2005;309(5734):626–30. Epub 2005/06/18. 10.1126/science.1112178 . 15961632

[51] Mavrich TN, Ioshikhes IP, Venters BJ, Jiang C, Tomsho LP, Qi J, et al A barrier nucleosome model for statistical positioning of nucleosomes throughout the yeast genome. Genome Res. 2008;18(7):1073–83. Epub 2008/06/14. 10.1101/gr.078261.108 . 18550805 PMC2493396

[52] Yuan GC, Liu JS. Genomic sequence is highly predictive of local nucleosome depletion. PLoS computational biology. 2008;4(1):e13 Epub 2008/01/30. 10.1371/journal.pcbi.0040013 . 18225943 PMC2211532

[53] Sekinger EA, Moqtaderi Z, Struhl K. Intrinsic histone-DNA interactions and low nucleosome density are important for preferential accessibility of promoter regions in yeast. Molecular cell. 2005;18(6):735–48. Epub 2005/06/14. 10.1016/j.molcel.2005.05.003 . 15949447

[54] Yazdi PG, Pedersen BA, Taylor JF, Khattab OS, Chen YH, Chen Y, et al Nucleosome Organization in Human Embryonic Stem Cells. PloS one. 2015;10(8):e0136314 10.1371/journal.pone.0136314 . 26305225 PMC4549264

[55] Liu MJ, Seddon AE, Tsai ZT, Major IT, Floer M, Howe GA, et al Determinants of nucleosome positioning and their influence on plant gene expression. Genome Res. 2015;25(8):1182–95. 10.1101/gr.188680.114 . 26063739 PMC4510002

[56] Mavrich TN, Jiang C, Ioshikhes IP, Li X, Venters BJ, Zanton SJ, et al Nucleosome organization in the Drosophila genome. Nature. 2008;453(7193):358–62. Epub 2008/04/15. 10.1038/nature06929 . 18408708 PMC2735122

[57] Nocetti N, Whitehouse I. Nucleosome repositioning underlies dynamic gene expression. Genes Dev. 2016;30(6):660–72. 10.1101/gad.274910.115 . 26966245 PMC4803052

[58] Lantermann AB, Straub T, Stralfors A, Yuan GC, Ekwall K, Korber P. Schizosaccharomyces pombe genome-wide nucleosome mapping reveals positioning mechanisms distinct from those of Saccharomyces cerevisiae. Nat Struct Mol Biol. 2010;17(2):251–7. 10.1038/nsmb.1741 . 20118936

[59] Valouev A, Johnson SM, Boyd SD, Smith CL, Fire AZ, Sidow A. Determinants of nucleosome organization in primary human cells. Nature. 2011;474(7352):516–20. Epub 2011/05/24. 10.1038/nature10002 . 21602827 PMC3212987

[60] Albert I, Mavrich TN, Tomsho LP, Qi J, Zanton SJ, Schuster SC, et al Translational and rotational settings of H2A.Z nucleosomes across the Saccharomyces cerevisiae genome. Nature. 2007;446(7135):572–6. 10.1038/nature05632 . 17392789

[61] Whitehouse I, Rando OJ, Delrow J, Tsukiyama T. Chromatin remodelling at promoters suppresses antisense transcription. Nature. 2007;450(7172):1031–5. 10.1038/nature06391 . 18075583

[62] Nalabothula N, McVicker G, Maiorano J, Martin R, Pritchard JK, Fondufe-Mittendorf YN. The chromatin architectural proteins HMGD1 and H1 bind reciprocally and have opposite effects on chromatin structure and gene regulation. BMC Genomics. 2014;15:92 10.1186/1471-2164-15-92 . 24484546 PMC3928079

[63] Andersson R, Enroth S, Rada-Iglesias A, Wadelius C, Komorowski J. Nucleosomes are well positioned in exons and carry characteristic histone modifications. Genome Res. 2009;19(10):1732–41. Epub 2009/08/19. 10.1101/gr.092353.109 . 19687145 PMC2765275

[64] Narlikar L, Gordan R, Hartemink AJ. A nucleosome-guided map of transcription factor binding sites in yeast. PLoS computational biology. 2007;3(11):e215 Epub 2007/11/14. 10.1371/journal.pcbi.0030215 . 17997593 PMC2065891

[65] Matys V, Fricke E, Geffers R, Gossling E, Haubrock M, Hehl R, et al TRANSFAC: transcriptional regulation, from patterns to profiles. Nucleic acids research. 2003;31(1):374–8. . 12520026 10.1093/nar/gkg108 PMC165555

[66] Small EC, Xi L, Wang JP, Widom J, Licht JD. Single-cell nucleosome mapping reveals the molecular basis of gene expression heterogeneity. Proceedings of the National Academy of Sciences of the United States of America. 2014;111(24):E2462–71. Epub 2014/06/04. 10.1073/pnas.1400517111 . 24889621 PMC4066511

[67] Baanannou A, Mojica-Vazquez LH, Darras G, Couderc JL, Cribbs DL, Boube M, et al Drosophila distal-less and Rotund bind a single enhancer ensuring reliable and robust bric-a-brac2 expression in distinct limb morphogenetic fields. PLoS genetics. 2013;9(6):e1003581 10.1371/journal.pgen.1003581 . 23825964 PMC3694829

[68] Couderc JL, Godt D, Zollman S, Chen J, Li M, Tiong S, et al The bric a brac locus consists of two paralogous genes encoding BTB/POZ domain proteins and acts as a homeotic and morphogenetic regulator of imaginal development in Drosophila. Development. 2002;129(10):2419–33. . 11973274 10.1242/dev.129.10.2419

[69] Rando OJ, Chang HY. Genome-wide views of chromatin structure. Annual review of biochemistry. 2009;78:245–71. Epub 2009/03/26. 10.1146/annurev.biochem.78.071107.134639 . 19317649 PMC2811691

[70] Zhang Y, Vastenhouw NL, Feng J, Fu K, Wang C, Ge Y, et al Canonical nucleosome organization at promoters forms during genome activation. Genome Res. 2014;24(2):260–6. 10.1101/gr.157750.113 . 24285721 PMC3912416

[71] Jin C, Zang C, Wei G, Cui K, Peng W, Zhao K, et al H3.3/H2A.Z double variant-containing nucleosomes mark 'nucleosome-free regions' of active promoters and other regulatory regions. Nature genetics. 2009;41(8):941–5. Epub 2009/07/28. 10.1038/ng.409 . 19633671 PMC3125718

