# Supplementary figures and images for: A comparison of nucleosome organization in Drosophila cell lines
Source: PLoS One. 2017 Jun 1;12(6):e0178590. doi: 10.1371/journal.pone.0178590 (PMC5453549; doi:10.1371/journal.pone.0178590)

## Supplemental Figure 1

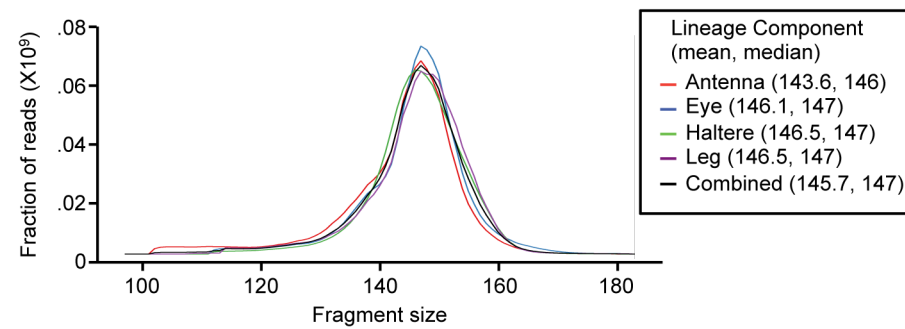

Supplement: S1 Fig — The distribution of the sequenced mononucleosomal DNA fragment lengths is very similar across all cell lines. (PDF) [file pone.0178590.s001.pdf]

# Supplemental Figure 2

**A**

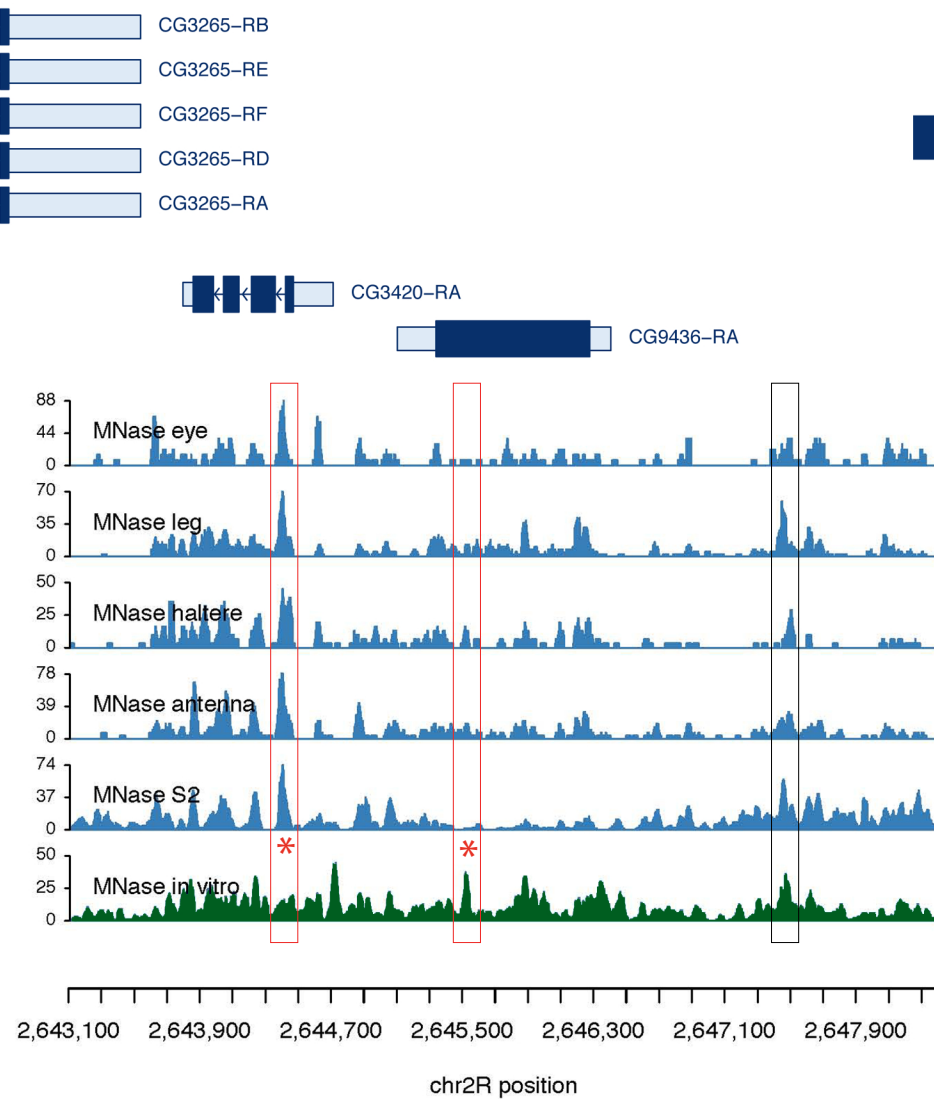

**B**

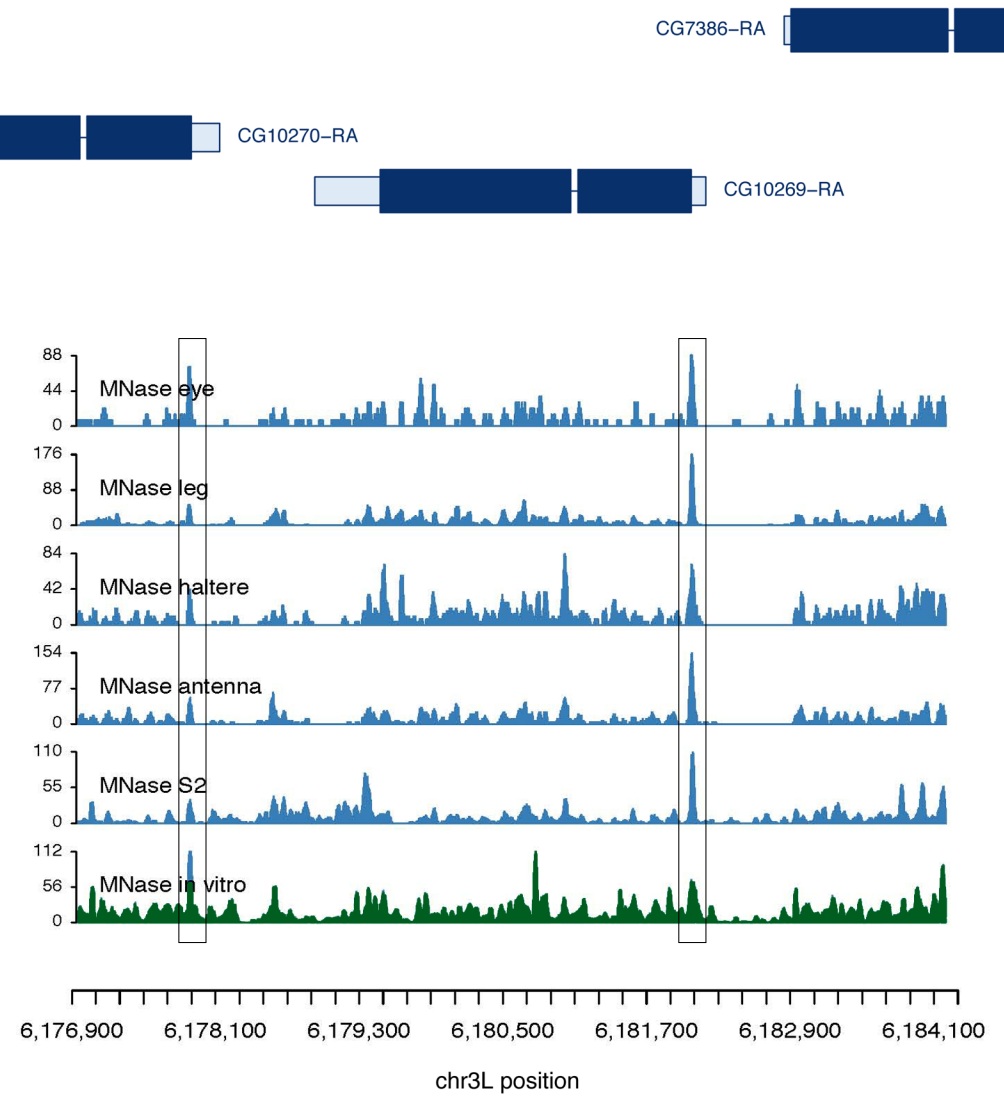

Supplement: S2 Fig — MNase midpoint density profiles, smoothed using a 30 bp sliding window, along randomly chosen genomic regions demonstrate that the nucleosome arrays from each cell line (top four tracks) correspond well with one another. The in vitro nucleosome arrays from this study (green, bottom tracks) correspond least well with nucleosome arrays generated from cells but maintain similar spacing and many of the strong and intermediate peaks. Example peaks that are similar between the cell lines and in vitro reconstituted chromatin are indicated with black boxes, while example peaks that are strong in all cell lines but reduced in in vitro chromatin, or vice versa, are shown in red boxes and marked with an asterisk. (PDF) [file pone.0178590.s002.pdf]
